# THE RELATION BETWEEN DEATH PREPARATION AND LIFE SATISFACTION AMONG KOREAN OLDER ADULTS

**DOI:** 10.1093/geroni/igac059.2138

**Published:** 2022-12-20

**Authors:** Suha You, Giyeon Kim

**Affiliations:** Chung-Ang University, Seoul, Seoul-t'ukpyolsi, Republic of Korea; Chung-Ang University, Seoul, Seoul-t'ukpyolsi, Republic of Korea

## Abstract

**Objectives:**

There is a growing interest in death preparation as an important process to achieve ego integrity at the last stage of life. However, in South Korea, conversations about death remain taboo especially with older adults. Thus, this study aims to examine the association between preparation for one’s death and life satisfaction among Korean older adults.

**Methods:**

Data for this study were drawn from the 2020 National Survey of Older Koreans conducted by the Korean Ministry of Health and Welfare. The sample was limited to adults aged 65 and older (N=10,097). The survey measured preparation for death, life satisfaction, and various covariates (e.g., sociodemographic characteristics, physical conditions, psychological conditions). Hierarchical multiple regression analysis was used to examine whether death preparation influences life satisfaction among Korean older adults.

**Results:**

Results from hierarchical multiple regression showed that after adjusting for covariates, those who reported more preparations for death mentally (B=.054, p<.001) and materially (B=.035, p<.001) were more likely to have greater satisfaction in life than their counterparts.

**Conclusion:**

Findings suggest that it is necessary to generate proper information and recognition about death at the government level and activate discourses on death preparation in later life.

